# Mobile Narcotic Treatment Programs: On the Road Again?

**DOI:** 10.7759/cureus.23221

**Published:** 2022-03-16

**Authors:** Frank Breve, Lisa Batastini, Jo Ann K LeQuang, Gina Marchando

**Affiliations:** 1 Department of Pharmacy, Temple University, Philadelphia, USA; 2 Legal Department, Mid Atlantic PharmaTech Consultants, LLC, Ventnor City, USA; 3 Pain Department, NEMA Research, Inc., Naples, USA; 4 Trauma Department, Summit Behavioral Health Center, Seabrook, USA

**Keywords:** opioid use disorder, opioid rehabilitation, mobile narcotic treatment program, mobile methadone clinic, methadone, buprenorphine

## Abstract

Many Americans with opioid use disorder (OUD), do not have access to treatment. Mobile narcotic treatment programs are now under new regulations that may make treatment more accessible to more people. These mobile programs can help expand the reach of opioid agonist treatment for OUD, help reduce human immunodeficiency viruses (HIV) and hepatitis C in the OUD population, and have retention rates that are often better than those at fixed-site clinics. Mobile services can also help reach marginalized individuals, the homeless, rural communities, and other underserved communities. They may offer methadone or buprenorphine treatment. Such mobile services have been used inside and outside the United States with promising results. In particular, mobile programs can make treatment available to people who do not have insurance, who lack reliable transportation, live in chaotic situations, or may be undomiciled. The potential pairing of mobile programs together with technology, such as smartphone apps or online resources, may allow mobile patients to benefit from counseling as well. Mobile clinics must be attached to a fixed-site narcotic treatment program and may have limitations with respect to the geographic area served. Mobile programs must have policies and procedures to store, transport, deliver, account for, reconcile, and dispose of opioid waste and would be subject to audit. Mobile opioid agonist therapy is an important and innovative service of particular value to underserved communities.

## Introduction and background

Only about 11% of the 19.9 million Americans with substance-use disorders received treatment in 2016 [1]. Barriers to treatment are numerous, but accessibility and affordability top the list. For people with opioid use disorder (OUD), opioid treatment with buprenorphine or methadone can aid in recovery, which must be viewed as a long-term process characterized by relapse [2]. Opioid agonist therapy requires the patient to take the drug daily in order to prevent withdrawal symptoms, reduce drug cravings, and eventually overcome or at least better manage their OUD [3]. Opioid agonist therapy confers many benefits to the patients, public health, and the community: lower rates of human immunodeficiency viruses (HIV) infections, hepatitis C, and relapse [4,5]. In optimal situations, opioid agonist treatments with buprenorphine or methadone are provided in tandem with counseling, psychological services, or other support networks [6]. A challenge in delivering opioid agonist treatment is the fact that people must report daily to the clinic, which can be a hardship if the clinic is distant, transportation is unavailable, or if the clinic hours do not align well with the individual’s work schedule [7]. Individuals sometimes miss their scheduled dose or even a series of doses, and this is alarming since such individuals may seek illicit opioids to manage their withdrawal symptoms and cravings. If they miss doses, they may have lost tolerance to opioids, making them more vulnerable to a potential overdose. Furthermore, when people are unable to get their daily treatment, there is a risk that they may turn to illicit street drugs to stave off withdrawal symptoms.

For decades, mobile methadone clinics have used vans or other vehicles to bring methadone maintenance programs into the community. The Drug Enforcement Administration (DEA) put a halt to approving new mobile programs in 2007 because of concerns about the diversion of controlled substances, but recently updated guidance about mobile narcotic treatment programs (MNTPs). This is welcome news because opioid overdose deaths are at record high levels [8], the pandemic has restricted movement in some communities, and there are no other treatment options for many people with OUD. The purpose of this article is to explore the emerging concept of mobile methadone maintenance therapy (MMT).

The authors searched PubMed for literature using the following keywords: “mobile methadone clinic” (32 results), “mobile methadone” (98 results), and “mobile narcotic treatment program (63 results). Google Scholar was searched as well. The Federal Register was also used to extract the legal text related to the new DEA regulation. This is a narrative review.

## Review

Short history of mobile narcotic treatment programs

Mobile narcotic treatment programs (MNTPs) dispensing methadone, nicknamed “methadone vans,” were being used for three decades, when in 2007, the DEA refused to license any new such mobile programs, citing concern over potential drug diversion [9]. However, since diversion did not seem to be a problem particular to mobile units, the reasoning behind this ban was not clear [10]. At present, there are 1700 DEA-registered brick-and-mortar opioid treatment facilities, some of which have a companion mobile service [11]. Effective on July 28, 2021, after considerable pressure from numerous organizations, the DEA issued new regulations, captured in 21 CFR (Code of Federal Regulations) Parts 1300, 1301, and 1304, related to registration requirements for narcotic treatment programs (NTP) with mobile components [12]. Under these new rulings, the MNTP must be part of a registered, fixed-site narcotic treatment program, and the mobile service may only operate in the state where the treatment program is registered. The mileage limits for the mobile unit are not specified, but it must return to a fixed site at the end of each day, where it must unload and store all controlled substances, and the vehicle must be parked in a secured, fenced location. Controlled substances placed in schedules II-V can be dispensed by an MNTP for maintenance or detoxification and do not require separate registration for the mobile component [12].

When these MNTPs or “methadone vans” were first rolled out in the United States, an important objective of theirs was to help bring these treatments to rural communities and underserved urban areas [13].

The value of opioid agonist treatment for OUD

Opioid agonist treatment using methadone has been recognized in a Cochrane review as being an effective treatment intervention for heroin dependence, decreasing heroin use to a greater extent than no treatment [14]. Likewise, buprenorphine treatment for maintenance in patients with OUD has been shown in a systematic review and meta-analysis to be similarly effective as methadone [15].

Since OUD is a long-term and even lifelong disorder, program retention rates are very important. Relapse is common among people with OUD; in fact, OUD is described as a chronically relapsing condition [5]. Retention rates are high in mobile programs [13]. In a study of homeless veterans (n=36), mean retention in mobile treatment was 19.2 months [16]. A study of 399 mobile narcotic treatment patients in Baltimore compared to 1588 patients at six fixed-site programs in the same area found that mobile patients had a median retention of 15.5 months compared to 3.9 months for fixed-site patients [17]. Furthermore, mobile programs have had similar retention rates outside the United States. For instance, a mobile methadone service in urban India found improved retention with the mobile programs compared to the fixed-site programs [18].

The value of mobile narcotic treatment programs

So-called “low-threshold programs” are designed to make OUD treatment as easy, straightforward, and accessible as possible, even for marginalized and hard-to-reach populations [19]. Low-threshold programs often must use technology, creativity, versatility, and innovation to reach their target populations. Conversely, high-threshold programs are defined as those that require abstinence. Medium-threshold programs are typically offered through healthcare providers along the lines of other drug prescriptions with weekly or biweekly office visits [19]. Not all people with OUD can succeed in a high-threshold program, and many may not have access to a medium-threshold alternative. In many cases, MNTPs are forms of low-threshold treatment programs, but they could be implemented in such a way as to encourage people who participate first in an MNTP outreach to avail themselves of higher-threshold programs in the future.

Opioid agonist treatment is available to only a subset of the 3 million Americans with OUD [20], and around the world, the proportion of those with OUD and access to treatment may be even lower. The barriers to OUD treatment are numerous and likely global: living too far from a clinic, lack of reliable transportation, lack of healthcare coverage, lack of funds, work or home schedules in conflict with clinic schedules, chaotic living situations, comorbidities such as mental health disorders, lack of awareness about opioid agonist treatment, stigmatization, and lack of knowledge as to how to avail such services. Thus, mobile programs may help to address some of these barriers.

In some nations in Europe, retail pharmacies are allowed to prescribe and dispense methadone to treat OUD [21]. Europe, Australia, and Thailand allow primary care physicians to dispense methadone for OUD [21]. While both of these strategies would make OUD treatment more readily accessible, neither of these options is legal in the United States.

Bringing treatment to the patient instead of the other way around may be another way to improve uptake of opioid agonist treatment. In a study of 539 methadone patients in China, 79.6% of patients had at some point interrupted or discontinued their methadone therapy, and one reason for this was the inability to find regular and reliable treatment at a fixed-site clinic [22]. Individuals with severe OUD may live in financially strained, chaotic conditions without access to a vehicle. A study of 7,918 U.S. census tracts found the median drive time to a fixed-site opioid treatment facility increased significantly from 2017 to 2018, from 16.1 minutes (range: 10.2-25.9) to 48.4 minutes (range: 34.0-63.3), p<0.001, and rural citizens were particularly hard hit with the long drive times [23]. For reasons not fully elucidated, various geographical regions of the United States have widely differing rates of OUD, with rural Appalachia as well as urban New England particularly hard hit [24]. While addiction is a problem in big cities, it has spread to the suburbs, small towns, and rural communities. In the United States, about 90% of all opioid treatment facilities are located in the city, leaving rural populations vastly underserved [10].

Mobile services may appeal to a broader and harder-to-reach patient base. In New Jersey, MNTP services were compared to fixed-site methadone clinics in the same areas. The mobile services tended to enroll more African-Americans, homeless people, individuals without insurance, injection drug users, daily drug users, and those who haven’t been in treatment recently [25]. This suggests that mobile services would likely benefit the marginalized, the disenfranchised, the underprivileged, and those who might lack access to a fixed-site clinic. People with OUD who were homeless or had instability with their domicile were shown in a study in Massachusetts to have lower retention in fixed-site opioid programs than those with more secure housing [26].

The experience in Amsterdam may be helpful in shaping our approach to this MNTP revitalization. About 30 years ago in Amsterdam, MNTP vans circulated the city with six daily stops where they dispensed methadone in a differentiated way [27]; for example, sex workers and those with a longer history of drug use received higher doses of methadone than those who were currently under the care of a physician [28]. A central methadone registry was set up to prevent people from obtaining multiple doses of methadone [29]. These methadone vans in Amsterdam maintained a list of registered clients, each of whom had been seen at a fixed-site clinic or health center for assessment and by a physician to set up their methadone doses. The van itself was run by a nurse and physician who treated only those patients on the registry, documenting all interactions. In Amsterdam, patients could advance over time from daily doses of methadone delivered by the van to weekly clinic visits with take-home oral medications [19]. However, Amsterdam did not require its methadone van clients to have any contact with a counselor or to be in any sort of recovery program. The use of illicit drugs by patients while on the program could not be used to exclude them. This program is still in operation today. Furthermore, all information about the methadone registry was and is today protected data under strict Dutch healthcare privacy laws [29].

Mobile treatment programs are intended to expand the reach of narcotics treatment programs and allow more people to utilize these programs. These programs may reduce the stigma of opioid rehabilitation and normalize such programs in communities, and it could also improve access to treatment for pregnant and postpartum individuals [30]. Mobile programs may be particularly relevant for homeless communities.

Mobile narcotic treatment programs now and in the future

Even before the pandemic brought a boom to telehealth interventions, technological advancements were seen as beneficial for those dealing with substance-use disorders [31]. Technology-based interventions, such as mobile phone contacts, video conferencing, text reminders, and app-based support, may provide specific benefits to OUD patients who may want the convenience of remote access, round-the-clock accessible support, and privacy [32]. Such innovations could readily be combined with mobile treatment, and OUD patients appear to support the use of technology in treatment [33]. The combination of internet-based counseling or online treatment tools in concert with opioid agonist therapy delivered by a mobile service holds great promise.

The main obstacles confronting the broader utilization of MNTPs are logistical. Clearer guidance and regulations from the DEA need to be issued. Since most of the mobile programs currently in operation are older, it is important to understand the sort of updates that are needed or that would be beneficial.

Fixed-site narcotics treatment programs registered with the DEA that wish to run a mobile unit must first outfit a suitable vehicle. Most importantly, the vehicle must have an integral secure safe for storage of controlled substances, and this safe must not be in any way accessible to anyone outside the van. This safe must be connected to an alarm that signals a centralized monitoring center and/or police. All controlled substances are to be transferred from the narcotic treatment program fixed-site facility before the van begins its day, and they must be brought back into the facility at the end of the run each day. As with any controlled substances, a system must be in place that allows for meticulous recordkeeping, reconciliation, and auditing. Facilities operating such mobile services must adhere to guidance about how to store and dispense the controlled substance and how to properly dispose of unused opioids. The vans must be set up to allow workspace for the clinician(s) and Wi-Fi access so that computers can be used for recordkeeping. Adequate privacy and security must be maintained.

Narcotic treatment programs must notify the DEA in writing of their intent to start a mobile narcotics treatment program and may operate only in the state(s) where the narcotic treatment center is registered. Further, the DEA must grant written approval to the narcotics treatment program before such a program can begin. Once in operation, a mobile narcotic treatment unit may not reverse-distribute, share, or transfer controlled substances from itself to another mobile unit away from the registered site of the narcotic treatment program. The mobile units are also not permitted to act as hospitals, long-term care facilities, or transport patients.

The narcotic treatment centers that run such programs must develop policies and procedures for safe, secure, and compliant operation. There must also be determinations as to the personnel and their training that run these vans, the hours of operation, patient registration, geographical areas to be covered, scheduling, and budget. The MNTP must develop their policies and procedures in alignment with the DEA, Food and Drug Administration, and Environmental Protection Agency for the appropriate handling, dispensing, accounting, and disposal of controlled substances. These procedures must, in particular, set forth how controlled substances are to be accounted for, how they are transferred between the treatment center and the mobile unit, and what will happen if, for some reason, the mobile unit is not able to operate. In addition, the service must commit to specific schedules and have trained individuals who can assure a robust chain of custody for these narcotics.

Storage and security emerge as primary considerations in any MNTP. Each MNTP vehicle must keep controlled substances in a securely locked safe in the vehicle equipped with an alarm system. This safe cannot be accessible from outside of the mobile unit. Patients who receive medication from a mobile unit must wait and remain in an area that is physically separated from the safe where the controlled substances are stored and the area where they are dispensed. This separation may be accomplished by a wall with a pass-through window, a door, or a seating area separated from the storage and dispensing area.

A challenge to all opioid agonist treatment remains the exit strategy for the patient. Many individuals embarking on rehabilitation will relapse, and some may discontinue opioid agonist therapy for good. Some relapse and return. Others persist in the treatment but never wean themselves entirely off opioids. With advances in healthcare and the ability to “manage” once fatal conditions, there is an older and aging population on opioid agonist therapy who enter their geriatric years as active substance abusers [34]. This older population may be well served by mobile treatment units. Furthermore, few programs aim at the aging substance abuser, many of whom have comorbid mental health issues that are also not frequently addressed in geriatric care [34]. There is no legally defined age limit for this service.

Legal considerations

**Figure 1:**
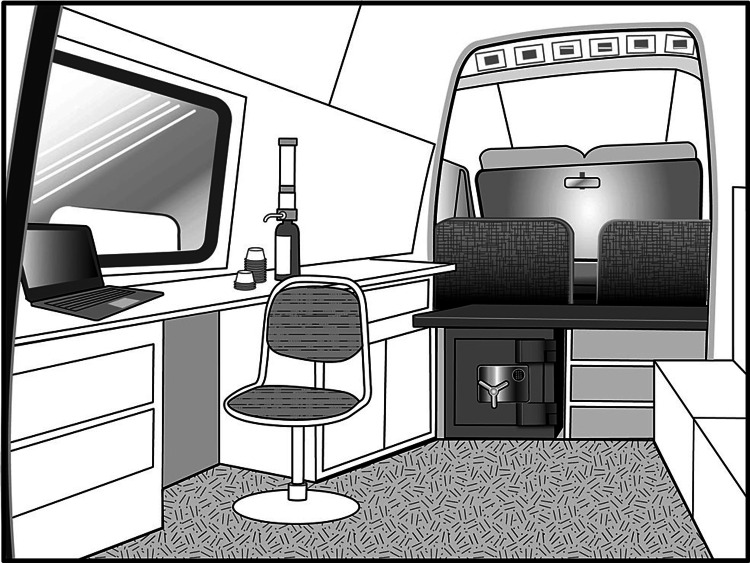
The interior of a mobile narcotic treatment van must utilize space efficiently. In this case, a pass-through window allows the clinician to treat patients, and the controlled substances safe is located behind the driver’s seat, making it inaccessible to the patients.

While MNTPs are both old and new, there are already known legal considerations in setting up such a program. Mobile units are required to keep a detailed log on the controlled substances they dispense, the doses, and the patients. If the records are electronic, a hard copy has to be printed out each day and initialed by the physician(s) who dispensed the controlled substances [10]. In addition to storing narcotics in a locked safe entirely within the vehicle, one person must be responsible for transporting and dispensing the controlled substances, which must be kept locked in the safe except when being dispensed. Patients must not be allowed to have access to the safe, either from outside the vehicle or while receiving their medication (see Figure 1).

There are no mileage restrictions on a mobile unit, except that it must return to the NTP base at the end of dispensing each day, and all controlled substances must be removed from the mobile unit for proper storage at its home narcotic treatment program. It is possible for programs to apply for a waiver to the DEA to allow for alternate return policies, but such decisions are made on a case-by-case basis. The fixed-site narcotic treatment program must park the vehicle in a secure, fenced parking area at the end of the day, and the DEA must be informed of the parking location. When the van is parked, no controlled substances should be kept in the safe.

## Conclusions

More and more accessible treatment options must be made available to the millions of people around the world suffering from OUD. MNTPs may help reduce some of the barriers to opioid agonist therapy, although their initiation requires investment and commitment on the part of a fixed-site narcotic treatment program. The risks of expanding mobile narcotic treatment units involve the security of the controlled substances, robust chain of custody, proper recordkeeping, appropriate dispensing, environmentally safe waste disposal, and audits. The DEA has set forth general guidelines and expects each mobile unit to be affiliated with a narcotic treatment program that will develop and adhere to policies and procedures. Mobile treatment vans are not inexpensive and must be specially outfitted with a separate waiting area for patients and a safe for storage of controlled substances. Mobile treatment programs may also be combined with smart technology, apps, and online services to enhance treatment. Mobile programs hold great promise to extend the reach of opioid agonist treatment to hard-hit communities.
